# Mapping the flow of painterly gesture

**DOI:** 10.1016/j.patter.2026.101516

**Published:** 2026-03-13

**Authors:** Lizhen Zhu, Chaewan Chun, Kathryn Brown, James Z. Wang

**Affiliations:** 1 Department of Informatics and Intelligent Systems, College of Information Sciences and Technology, The Pennsylvania State University, University Park, PA 16802, USA; 2 Department of Communication and Media, School of Social Sciences and Humanities, Loughborough University, Loughborough, LE11 3TU, UK

## Abstract

We introduce streamline-based visualizations that manifest the detailed directional structures of painted surfaces by integrating local gradient information into continuous flow representations. Using Impressionist paintings as a primary case study, we position these visualizations as interpretive instruments that render the gestural production of artworks perceptible, comparable, and accessible to both scholars and non-specialist audiences.

## Main text

Impressionist paintings are noted for their luminous, texturally rich surfaces, and bold brushwork plays a central role in shaping their visual impact and meaning. Although styles differed between painters, Impressionist artists typically privileged immediacy over finish, allowing a wide variety of gestures to remain a visible and assertive presence in the final work. Despite its significance, the directional organization of brushstrokes remains difficult to isolate or analyze systematically in individual paintings. Here, we investigate whether streamlines, derived from local gradients and directional cues embedded in the painted surface, can offer a computational “mapping” of painterly gesture through the manifestation of brushstroke directionality.

### Transforming local cues into painterly flow

Our approach extends earlier work on localized regions in Claude Monet’s *Water Lilies*^1^ to operate at the scale of entire paintings, enabling the visualization of directional organization across the full pictorial surface. Local gradients are computed with Sobel operators^2^ and encoded via a structure tensor^3^ to capture orientation and directional coherence. The tensor field is smoothed to reduce pixel-level noise while preserving painterly flow, yielding a continuous orientation field across the image.

Streamlines are traced from sampled seed points, extending forward and backward along dominant local directions while favoring directional continuity. Growth terminates when directional changes become abrupt, when local structure becomes isotropic, or when a streamline approaches an already detected path. Through this process, dispersed local cues are integrated into smooth trajectories that reveal coherent directional flows across the painted surface.

Following extraction, streamlines whose length exceed a threshold relative to the image size are selected to reduce visual clutter and emphasize spatially coherent flow. These streamlines are visualized in two complementary ways: overlaid directly on the original painting to preserve color and texture or rendered on a grayscale background with color encoding local orientation to make directional variation explicit.

### Directional flow as data-mediated gesture

**Figure 1:**
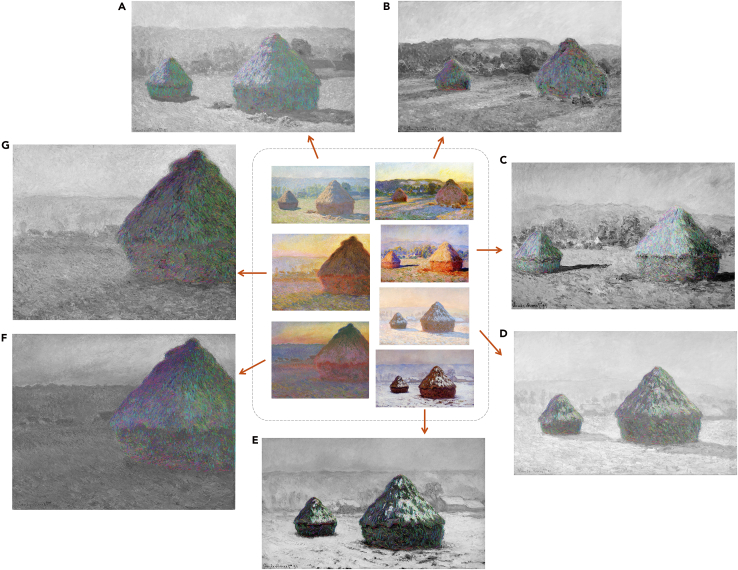
Streamlines extracted from Claude Monet’s *Haystacks* series Center: original oil-on-canvas paintings. Surrounding panels: colored streamlines over grayscale renderings of the same works. (A) *Stacks, End of Summer*, 1891, Musee d’Orsay, Paris, France. (B) *Stacks of Wheat (End of Summer)*, 1890–1891, Art Institute of Chicago, Chicago, IL, USA. (C) *Grainstacks in the Sunlight, Morning Effect*, 1890, private collection. (D) *Wheatstacks, Snow Effect, Morning*, 1891, The J. Paul Getty Museum, Los Angeles, CA, USA. (E) *Grainstack, White Frost Effect*, 1890–91, Shelburne Museum, Shelburne, VT, USA. (F) *Grainstack in the Sunlight*, 1891, private collection. (G) *Grainstack. (Sunset.)*, 1890–91, Museum of Fine Arts, Boston, MA, USA.

We examined works from Claude Monet’s *Haystacks* series to illustrate how directional flow encodes both volumetric structure and illumination. Streamlines traced over the central forms follow the conical geometry of the stacks, echoing the physical orientation of straw fibers and the sloping topography of the haystack itself. As shown in Figures 1F and 1G, streamline directions transition smoothly with changes in color, while across varying lighting conditions (Figures 1A–1E), their geometry responds systematically to illumination. On sunlit surfaces, directional flow opens outward, occasionally radiating toward the light source. By contrast, in shadowed regions it becomes more parallel and constrained. When the stacks are backlit (Figure 1B), the increased curvature delineates deep cast shadows, indicating that directional flow is indexed to light. This offers new insight into the relationship between Monet’s brushwork, his repeated experimentation with a central motif, and the rendering of different atmospheric conditions.

**Figure 2:**
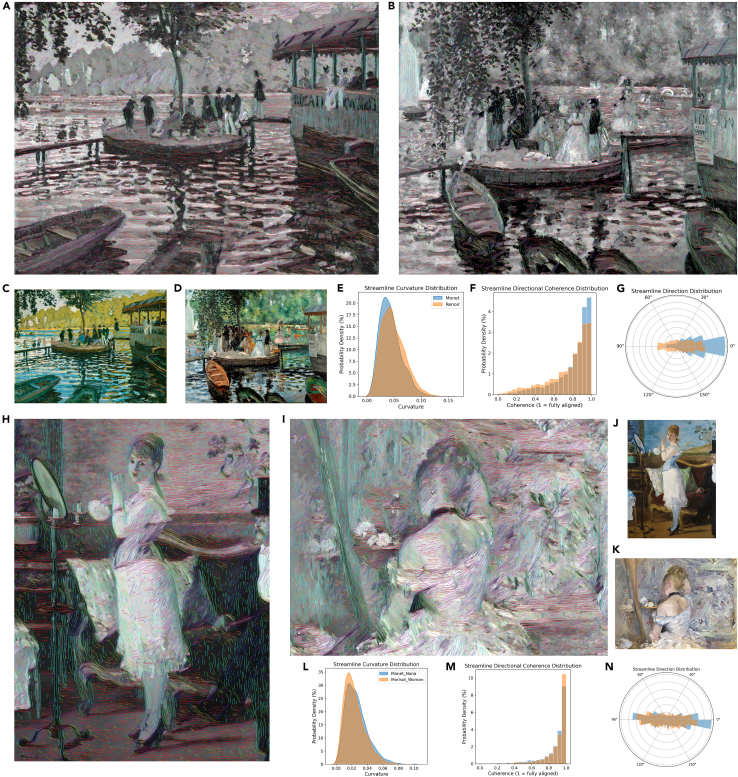
Qualitative visualization and quantitative comparison of streamlines in Impressionist paintings (A and B) Streamline detection results on Monet’s *La Grenouillère* (A) and Renoir’s *La Grenouillère* (B). (C) *La Grenouillère*, Claude Monet, 1869, oil on canvas, Metropolitan Museum of Art, New York City, NY, USA. (D) *La Grenouillère*, Pierre-Auguste Renoir, 1869, oil on canvas, Nationalmuseum, Stockholm, Sweden. (E–G) Comparative distributions of curvature (E), per-streamline orientation consistency (F), and mean orientation (G) for the works in (A) and (B). (H and I) Streamline detection results on *È*douard Manet’s *Nana* (H) and Berthe Morisot’s *Woman at Her Toilette* (I). (J) *Nana*, *È*douard Manet, 1877, oil on canvas, Hamburger Kunsthalle, Germany. (K) *Woman at Her Toilette*, Berthe Morisot, 1870–1880, oil on canvas, Art Institute of Chicago, IL, USA. (L–N) Comparative distributions of curvature (L), per-streamline orientation consistency (M), and mean orientation (N) for the works in (H) and (I).

To support comparative interpretation, we characterize streamlines by using length, curvature, average orientation, and directional coherence (Figure 2). These features enable comparisons across artists and stylistic approaches. For example, in Pierre-Auguste Renoir’s *La Grenouillère*, higher curvature and rapidly shifting directions reflect his use of short, broken, and swirling brushstrokes that produce a fragmented visual effect. By contrast, in Monet’s rendering of the same subject, streamlines exhibit greater directional consistency, corresponding to his more structured rendering of the water surface through longer, horizontal strokes. A similar contrast appears between another two works that treat a similar subject: Édouard Manet’s *Nana* and Berthe Morisot’s *Woman at Her Toilette*. While Manet was noted for his vivid mark-making in the nineteenth century, *Nana* displays passages with consistently oriented brushstrokes that impose a strong internal structure on the composition. In comparison to this approach, the highly varied directional flow in Morisot’s canvas casts the paint surface as a contested field in which form threatens to dissolve in a dynamic, atmospheric field of gestures.

### Interpretive significance

By transforming local observations into continuous visual structures, these visualizations provide an intricate map of a painting’s unique surface that supports closer analysis of gestural practice, compositional organization, and stylistic variation. We frame this approach not as a definitive account of pictorial structure, but as a computationally grounded interpretive tool for evaluating how artworks communicate through the detailed directional flow of their brushwork. The resulting visualizations open fresh perspectives on the ways in which motion, pressure, and repetition accumulate over a painted surface, highlighting large-scale coherence and/or areas of localized disruption. While this is important for the interpretation of individual paintings, it also has the capacity to challenge monolithic approaches to the definition of artistic movements that subsume different works under the umbrella of a dominant style or method of paint handling. Put another way, the proposed visualizations add a new layer of information to the stock of data available to art historians and facilitate a more nuanced approach to interpreting different creative practices.

**Figure 3:**
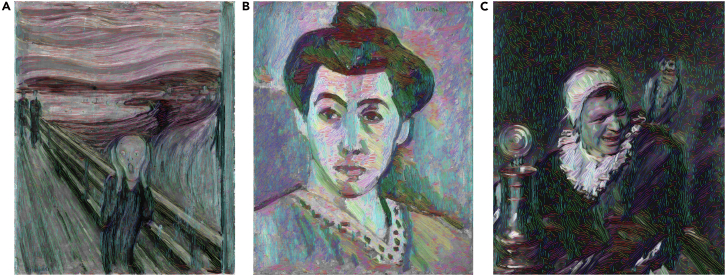
Streamlines extracted from diverse artworks, illustrating directional flow across styles Color encodes local streamline orientation, making directional variation explicit. (A) *The Scream*, Edvard Munch, 1893, tempera and crayon on unprimed cardboard, ProtoExpressionism, Munch Museum, Oslo, Norway. (B) *Portrait of Madame Matisse*. *The Green Line*, Henri Matisse, 1905, oil on canvas, Fauvism, Statens Museum for Kunst, Copenhagen, Denmark. (C) *Malle Babbe*, Frans Hals, 1633–1635, oil on canvas, Dutch Golden Age, Gemäldegallerie, Berlin, Germany.

Impressionist painting served as our primary case study because of the aesthetic importance that has traditionally been attached to the dynamic brushwork associated with this movement. However, the approach is not limited by genre. As illustrated in Figure 3, streamlines reveal meaningful directional structures within works drawn from diverse artistic traditions and can open new conceptions of painterly processes and meaning-making that lie beyond the immediately visible features of a painted surface. This suggests that the method of computational visualization proposed here can function as an interpretive partner that complements qualitative research and expands how the underlying visual structures of paintings are perceived, communicated, and shared.

## Acknowledgments

The Penn State researchers were supported in part by the National Science Foundation (NSF) under award no. 2234195. Computational resources were provided through an allocation from the Advanced Cyberinfrastructure Coordination Ecosystem: Services & Support (ACCESS) program, also supported by NSF. K.B. thanks the British Academy for supporting the initial stages of this research (award no. TDA21:210060).

All paintings discussed in this work are in the public domain under US copyright law. The analyses are conducted on digital reproductions obtained from publicly accessible museum and library collections and from WikiArt.org as an image aggregation platform. Original artworks are shown only as reduced-resolution thumbnails, while higher-resolution figures contain computationally generated overlays produced by the research team. Full attribution to the source museums or collections is provided in the figure captions.

## Declaration of interests

The authors declare no competing interests.

## Declaration of generative AI and AI-assisted technologies in the writing process

During the preparation of this manuscript, the authors used ChatGPT-5.2 to assist with improving clarity and readability. All content was subsequently reviewed and edited by the authors, who take full responsibility for the accuracy, integrity, and originality of the work.
